# Un-biased housekeeping gene panel selection for high-validity gene expression analysis

**DOI:** 10.1038/s41598-022-15989-8

**Published:** 2022-07-19

**Authors:** Ana I. Casas, Ahmed A. Hassan, Quirin Manz, Christian Wiwie, Pamela Kleikers, Javier Egea, Manuela G. López, Markus List, Jan Baumbach, Harald H. H. W. Schmidt

**Affiliations:** 1 Department of Neurology and Center for Translational Neuro- and Behavioural Sciences (C-TNBS), University Clinics Essen, Essen, Germany; 2 Department of Pharmacology & Personalised Medicine, MeHNS, Faculty of Health, Medicine and Life Sciences, Maastricht University, Maastricht, The Netherlands; 3 Faculty of Mathematics, Informatics and Natural Sciences, University of Hamburg, Hamburg, Germany; 4 Department of Mathematics and Computer Science, University of Southern Denmark, Odense, Denmark; 5 Molecular Neuroinflammation and Neuronal Plasticity Research Laboratory, Hospital Universitario Santa Cristina, Instituto de Investigación Sanitaria-Hospital Universitario de la Princesa, Madrid, Spain; 6 Departamento de Farmacología, Instituto de I+D del Medicamento Teófilo Hernando (ITH), Facultad de Medicina, Universidad Autónoma de Madrid, Madrid, Spain; 7 Chair of Experimental Bioinformatics, TUM School of Life Sciences Weihenstephan, Technical University of Munich, Munich, Germany

**Keywords:** Computational biology and bioinformatics, Computational platforms and environments

## Abstract

Differential gene expression normalised to a single housekeeping (HK) is used to identify disease mechanisms and therapeutic targets. HK gene selection is often arbitrary, potentially introducing systematic error and discordant results. Here we examine these risks in a disease model of brain hypoxia. We first identified the eight most frequently used HK genes through a systematic review. However, we observe that in both ex-vivo and in vivo*,* their expression levels varied considerably between conditions. When applying these genes to normalise expression levels of the validated stroke target gene, inducible *Nox4*, we obtained opposing results. As an alternative tool for unbiased HK gene selection, software tools exist but are limited to individual datasets lacking genome-wide search capability and user-friendly interfaces. We, therefore, developed the HouseKeepR algorithm to rapidly analyse multiple gene expression datasets in a disease-specific manner and rank HK gene candidates according to stability in an unbiased manner. Using a panel of de novo top-ranked HK genes for brain hypoxia, but not single genes, *Nox4* induction was consistently reproduced. Thus, differential gene expression analysis is best normalised against a HK gene panel selected in an unbiased manner. HouseKeepR is the first user-friendly, bias-free, and broadly applicable tool to automatically propose suitable HK genes in a tissue- and disease-dependent manner.

## Introduction

Analysing differential gene expression via mRNA levels is a common tool in biomedical sciences to understand cellular regulation^1^ or its dysregulation in disease. One application is the identification of target genes for therapeutic intervention and drug discovery^2^. The gold standard and most widely used technology to quantify mRNA levels is real-time quantitative PCR (RT-qPCR) because of its high sensitivity and specificity^3^. However, to be able to draw a reliable conclusion with respect to differential expression, e.g., health *vs* disease or control *vs* treatment, normalisation to a stable so-called housekeeping gene (HK) is essential. For this purpose, it is common practice to select genes related to basal cell metabolism as their expression is thought to be stable and thus fulfil the key HK requirement^4^. However, the expression levels of several commonly used HK genes, in fact, strongly vary between different cell types, disease models and drug interventions^4,5^. Since the validity of a differential gene expression result^6^ resides entirely on the stability of the chosen HK gene, diametrically opposing results can be obtained if data normalisation is performed using unstable and differentially regulated HK genes^7,8^. Moreover, on top of a disease condition, therapeutic drugs may further modulate HK gene expression introducing further variability. In the worst scenario, such results, if used for cell-based diagnostics, e.g., in liquid biopsies, could mislead subsequent drug intervention.

To prevent such analytical errors, several mitigation methods have been suggested. The most basic approach is the comparative delta-Ct method ranking stably expressed genes based on pair-wise comparisons^9^. Other strategies use expression variance of either an individual gene, a subset of genes, or all genes represented as a co-variance matrix^10–12^. However, these options are based on the assumption of a constant composition of all samples, which, however, is not necessarily the case. Similarly, HK genes under different conditions were selected based on microarray data^13–15^, again assuming that the vast number of publicly available data sets resolves preselection bias. However, most of these methods were neither automated nor made available for future access^11,16^, with RefGenes^11^ and NormFinder^12^, and BestKeeper^10^ being three positive examples. However, even these three tools have significant limitations as they do not scale well for use in genome-wide de novo searches for HK gene candidates.

We here address these fundamental gaps in HK gene identification by developing a user-friendly, bias-free, and broadly applicable tool to automatically propose suitable HK genes, e.g., suitable for a given tissue and disease condition. We then validate this approach by comparing classical biased KH gene selection for brain hypoxia to a de novo identified highly stable HK panel and their reliability in reproducing the discovery of *Nox4* as a key inducible gene with a causal role in post-stroke neurodegeneration^17^. We thereby also address whether, for the analysis of differential gene expression, single HK genes suffice or HK gene panels are preferable.

## Results

### Systematic review on the most used HK genes in brain hypoxia

The selection of HK genes is often perpetuated from one publication to another^18^. We conducted a systematic literature review to identify the most frequently used HK genes in two broadly used ex vivo brain hypoxia models, rat hippocampal brain slices (HBS) and rat organotypic hippocampal culture (OHC). Specifically, 38 and 16 original articles were identified with both ex vivo animal models, respectively (Supplementary Fig. 1), with six potential HK genes of which *β-actin* and *Gapdh* were most frequently used (Table 1). Two additional genes, *Sdha* and *Ywhaz*^19^, were previously suggested as the most suitable for differential gene expression normalisation in vivo stroke models. These eight genes were then validated for stability in three widely used brain hypoxia models in two different species, ex vivo in rat HBS or OHC subjected to oxygen and glucose deprivation (OGD) and in vivo in the mouse middle cerebral artery occlusion (MCAO) model.

**Table 1:** Normalization genes used for in vitro gene expression determination in brain ischemia models.

Hippocampal brain slices—acute model								
Published	Excluded	Included	Genes					
364	353	11	*Hprt* 1	*β-actin* 4	*18S* 1	*Gapdh* 5		

Organotypic hippocampal culture—chronic model								
Published	Excluded	Included	Genes					
249	222	27	*β-actin* 9	*Hprt* 1	*Gapdh* 12	*18S* 2	*β2-mg* 2	*Rpl13* 1

**Figure 1:**
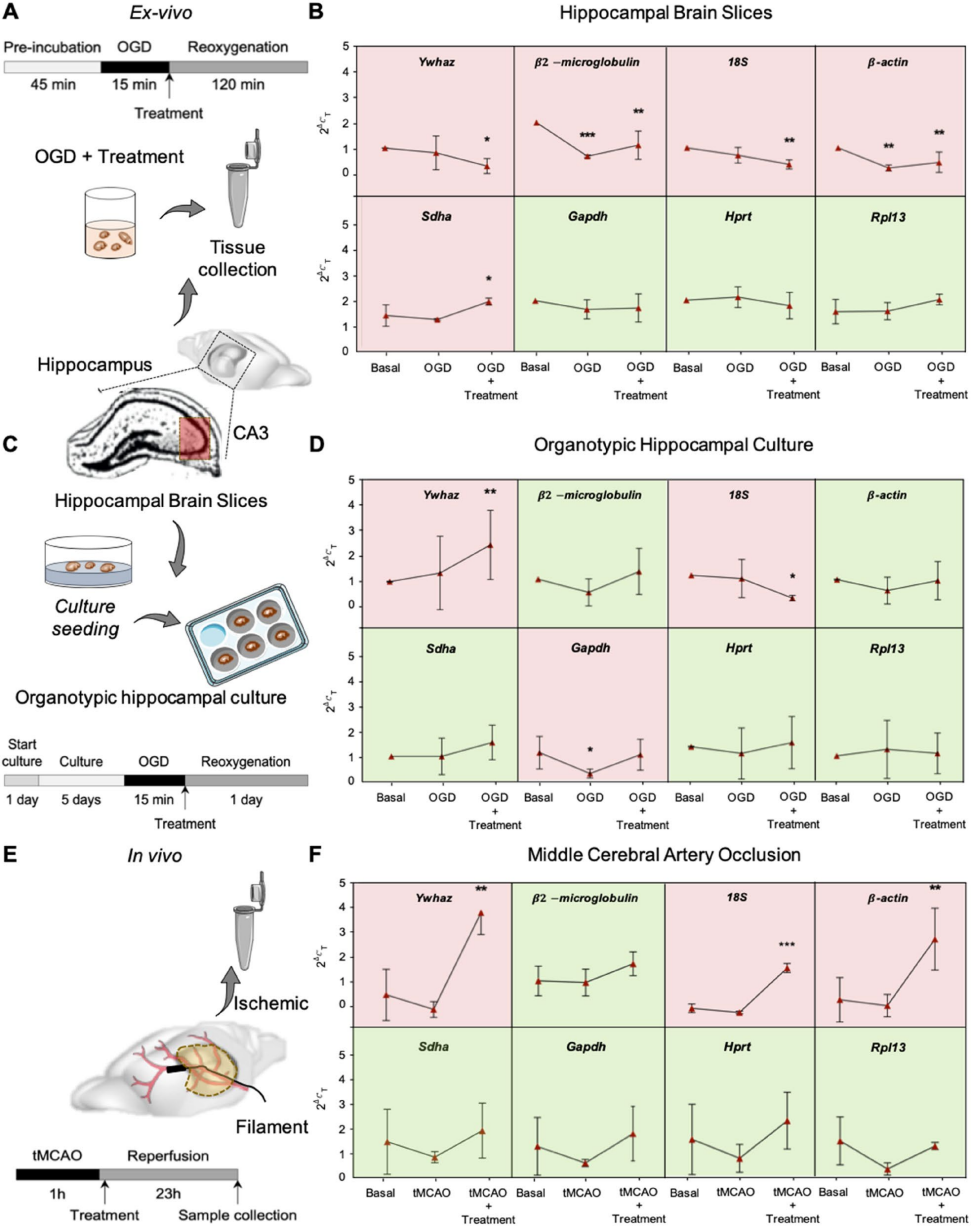
Experimental validation of eight housekeeping genes in three different brain ischemia models. (**A**) Rat hippocampal brain slices were stabilised for 45 min, followed by 15 min of oxygen and glucose deprivation (OGD) and 2 h of re-oxygenation (Re-Ox). (**B**) *Gapdh, Hprt* and *Rpl13* gene expression remains stable while *Ywahz, 18S* and *Sdha* are significantly up-or down-regulated after pharmacological intervention. *β2-microglobulin* and *β-actin* showed instability in all hypoxic conditions (*p < 0.05, **p < 0.01, ***p < 0.001, n = 5). (**C**) After 5 days of culture stabilisation, organotypic hippocampal cultures (OHCs) were exposed to 15 min of OGD followed by 24 h of Re-Ox. (**D**) While *β2-microglobulin, β-actin, Sdha, Hprt and Rpl13* expression remains stable under all experimental conditions, *Ywhaz, Gapdh and 18S* resulted in unsuitable housekeeping genes for this model (*p < 0.05, **p < 0.01, n = 5). (**E**) Adult WT mice were subjected to a 1 h-transient occlusion of the middle cerebral artery (tMCAO) followed by 23 h of reperfusion. (**F**) *β2-microglobulin, Sdha, Gapdh, Hprt and Rpl13* gene expression remained stable under all interventions while *Ywhaz, 18S* and *β-actin* are significantly up-regulated due to treatment (*p < 0.05, **p < 0.01, ***p < 0.001, n = 5).

First, rat HBS were subjected to OGD for 15 min, followed by 2 h of re-oxygenation (Re-Ox) with or without different pharmacological interventions (Fig. 1A). Upon RT-qPCR analysis (Supplementary Table 2), five of the eight presumable HK genes significantly varied in expression with or without OGD and were considered unsuitable for this model. Only *Gapdh*, *Hprt* and *Rpl13* remained stable (Fig. 1B). Second, OHCs were subjected to 15 min of OGD followed by 24 h of re-oxygenation with or without different pharmacological treatments (Fig. 1C). Here, three genes were unacceptable, *Ywhaz* and *18S*, but surprisingly, also the most widely used HK gene, *Gapdh* (Fig. 1D). Finally, adult mice were subjected to a 1 h transient MCAO with or without subsequent pharmacological treatments (Fig. 1E). In line with the previous ex vivo findings, *Ywhaz* and *18S* were too variable, but with *β-actin is* also one of the most frequently used HK genes in biomedicine (Fig. 1F). Thus, for brain hypoxia, HK genes are highly variable across different experimental models, species, and pharmacological interventions. Therefore, even for this single disease condition, no standard HK gene recommendation can be inferred from the literature, even for highly frequently used genes.

### Contradicting results on *Nox4* expression when using stable and rejected HK genes

To test the consequences of such HK gene variability for the analysis of differential gene expression, we used the established^20^ and repeatedly confirmed^21^ upregulation of NADPH oxidase 4 (*Nox4*) upon hypoxia as a test case. Upon brain hypoxia, *Nox4* is broadly induced in different cell types including blood–brain barrier cells and neurons^22^; genetic deletion of *Nox4* or pharmacological inhibition of the NOX4 enzyme is directly neuroprotective, stabilises the blood–brain barrier, and improves neuro-motor function^22^. Therefore, NOX4 could be considered a prominent therapeutic target in ischemic stroke.

**Figure 2:**
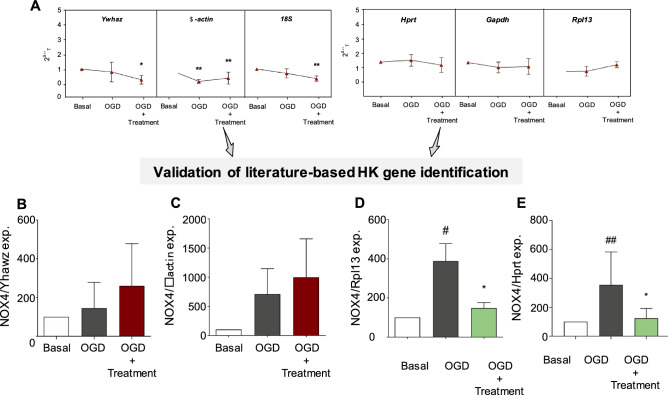
Opposing gene expression results are based on specific housekeeping gene selection. (**A**) HK gene validation using acute hippocampal brain slices (HBS) identified *Yhawz*, $\beta$*-actin*, and *18S* as non-stable, while *Hprt*, *Gapdh*, and *Rpl3* resulted in the best candidates for this model. (**B**) NADPH oxidase 4 (*Nox4*) gene expression was assessed in HBSs subjected to 3 different experimental conditions, (i) basal, (ii) oxygen and glucose deprivation (OGD), and (iii) OGD under treatment conditions. *Nox4* gene expression was normalised against previously detected unstable genes (*Yhawz*$, \beta$*-actin, and 18S*) and the most stable candidates (*Hprt, Gapdh, and Rpl13*). Diametrically opposing results were identified depending on the selected HK gene (^*#*^*p* < 0.05, ^*##*^*p* < 0.01 n = 5) later prevented by 2 h of pharmacological treatment (*p < 0.05, n = 5).

We analysed *Nox4* gene expression in all three above models of brain hypoxia with and without pharmacological treatment. For normalisation, we either used the three commonly used HK genes that we, however, rejected because of instability, i.e., *Yhwaz*, *β-actin*, and *18S*, or three HK genes that we confirmed or found to be sufficiently stable for these models, i.e., *Hprt*, *Gapdh*, and *Rpl13* (Fig. 2A). *Nox4* expressional normalisation using the latter three stable HK gene candidates resulted in the reproduction of a significant *Nox4* upregulation post hypoxia which was prevented by pharmacological intervention. In contrast, normalisation using the former unstable genes resulted in opposing results contradicting previous literature^23^ (Fig. 2B–E). Hence, identical therapeutic interventions would have been interpreted as either neuroprotective or ineffective depending on the gene used for expressional normalisation.

### HouseKeepR allows for robust de novo normalisation gene identification

Our literature-based HK identification approach shows that relying on previous publications, even in the same experimental model, is insufficient for HK gene selection and can lead to grossly misleading results. Thus, experimental validation of gene stability is essential per model, tissue, and condition, leading to additional experiments and, more importantly, would still be biased because the initial gene selection is only based on previous similar and published use. Potentially more stable and useful HK genes may be systematically missed by this approach.

**Figure 3:**
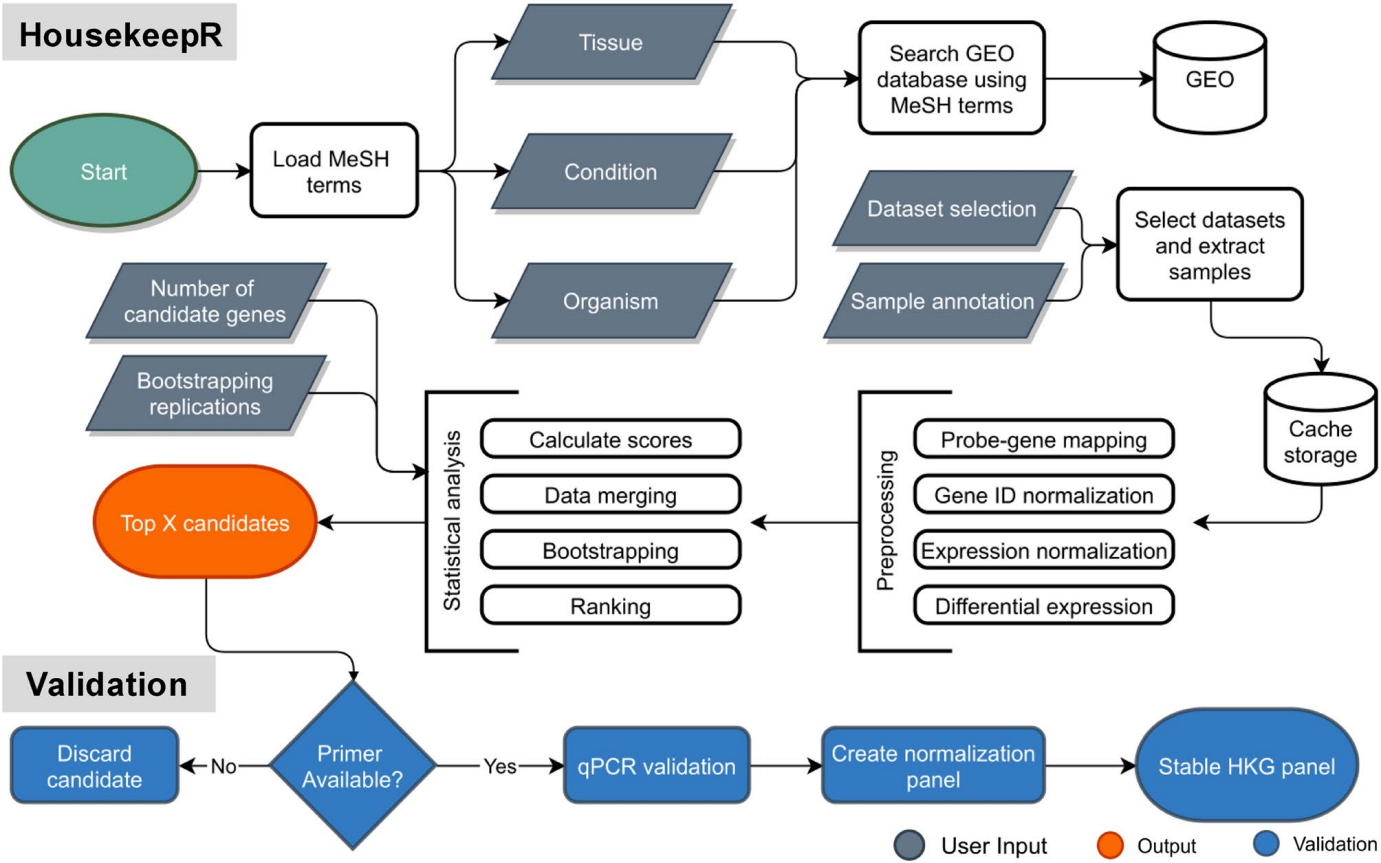
HouseKeepR algorithm flow chart. First, MeSH terms are parsed and preloaded to be used as standardised search terms for expressions data sets. User inputs for tissue, condition and organism, encoded in MeSH terms, are used to search and download data sets from Gene Expression Omnibus (GEO). The data sets are then pre-processed and statistically analysed using a scoring formula engineered to find normalisation genes. The top-ranking genes, based on user choice, are selected to be validated. In this scenario, normalisation genes within the first 25 ranked candidates were included in the subsequent validation process. Grey-colored elements show user required input, while the orange elements are the outputs of the user, and the blue elements are external validation steps conducted via wet-lab experiments.

To overcome this knowledge problem and the methodological gap in gene expression normalisation, we developed HouseKeepR, a web tool that robustly and rapidly ranks genes across relevant and publicly available expressional data sets (Fig. 3). Existing approaches for this task rank genes only by variance; HouseKeepR, also for consistently high average expression individually for each data set. Moreover, to ensure that results are robust towards sampling bias, HouseKeepR uses a bootstrapping strategy to obtain a distribution, expected mean rank, and rank variance for each gene.

### HouseKeepR is easily accessible through a user-friendly web interface

For user-friendliness, HouseKeepR can be applied to specified gene expression data sets, which are automatically retrieved from the public Gene Expression Omnibus (GEO) database^24^. Moreover, to encourage broad applicability also by non-bioinformaticians, we have developed a convenient web interface with integrated search and sample annotation functions (https://exbio.wzw.tum.de/housekeepr). HouseKeepR requires only three main input parameters, i.e., tissue, condition, and organism, to automatically identify the most suitable HK gene candidates. Optional parameters include the number of final HK gene candidates to be identified or the number of bootstrap replications (default is 10,000 repetitions).

HouseKeepR then searches the GEO and Ensembl databases and reports back all data sets matching the selected parameters^24^. Next, users must choose at least two of those data sets and annotate condition and control samples to perform the analysis. The server will then download these data sets and run the HouseKeepR algorithm. Finally, results are displayed as a ranking table (Supplementary Fig. 2) and a rank distribution across data sets (Supplementary Fig. S3). The run time depends on the number of data sets and the response time of the GEO database. Importantly, datasets are subject to updates, possibly affecting reproducibility over time. To address this issue, HouseKeepR allows users to save sessions, which preserve dated results and the parameters used for later retrieval. Furthermore, users can optionally select even older versions from the archive if required for specific questions. With this, HouseKeepR enables every biomedical researcher to quickly and easily identify stable normalisation genes for differential gene expression analysis via a meta-analysis for any tissue, condition, and organism benefiting from a large and continuously growing number of publicly available data sets.

### HK genes candidates are robust across non-overlapping data sets

As described, users have the option to select at least two or more databases for HK gene identification. Since different users may select different databases, we next examined whether the selection of non-overlapping databases may affect the robustness of HouseKeepR. Through bootstrapping, HouseKeepR allows studying the stability of the results under different random samplings from the included data sets. As additional consistency validation, we executed HouseKeepR 10 times with the same data sets and bootstrap parameters but using different random seeds. In this context, we computed the reproducibility measure (R) as the average of all pairwise overlap coefficients calculated between each pair of results lists (with the top 50 HK genes) produced by each run. The ten runs achieved an R-value > 0.99, which demonstrates the robustness of our algorithm. Another important measure for HouseKeepR is the reproducibility of HK gene candidates when comparing different non-overlapping data sets for the same tissue, organism and condition. To double-check this functionality, we selected two non-overlapping sets of 12 and two data sets, respectively. The top 20 HK gene candidates of both groups were compared, yielding an overlap coefficient of 0.5 (p-value = 9.75e−29) (Supplementary Fig. S4)^25^. The significant overlap indicates that HouseKeepR successfully predicts stable HK genes across different expression platforms and experimental setups. Contrary, as a negative control, we ran HouseKeepR on a group of two data sets of different organisms and conditions. Importantly, the overlap coefficient of the top 20 HK genes to the previously selected ones was 0 (Supplementary Fig. S4), confirming that predictions are condition- and organism-dependent, which underlines the necessity to use HouseKeepR for HK gene selection.

### NormFinder confirms the stability of HouseKeepR candidate genes

As mentioned, HouseKeepR is not the first software of its kind, although it has major advantages concerning usability and breadth of application. Nevertheless, we next cross-evaluated the results of HouseKeepR with a similar computational approach, NormFinder. The latter employs a more elaborate statistical model to test gene stability which is computationally expensive and thus not suited for systematic screening across several data sets (Supplementary Fig. 5). Stable genes were defined using NormFinder’s stability score with a cut-off value of 0.15). 80% of the top 10 HK genes (Supplementary Fig. 6) generated by HouseKeepR were confirmed to be stable by NormFinder, highlighting that HouseKeepR reported genes are also considered robust by independent statistical evaluation.

### HouseKeepR suggested HK genes are consistent in *Nox4* target validation

**Figure 4:**
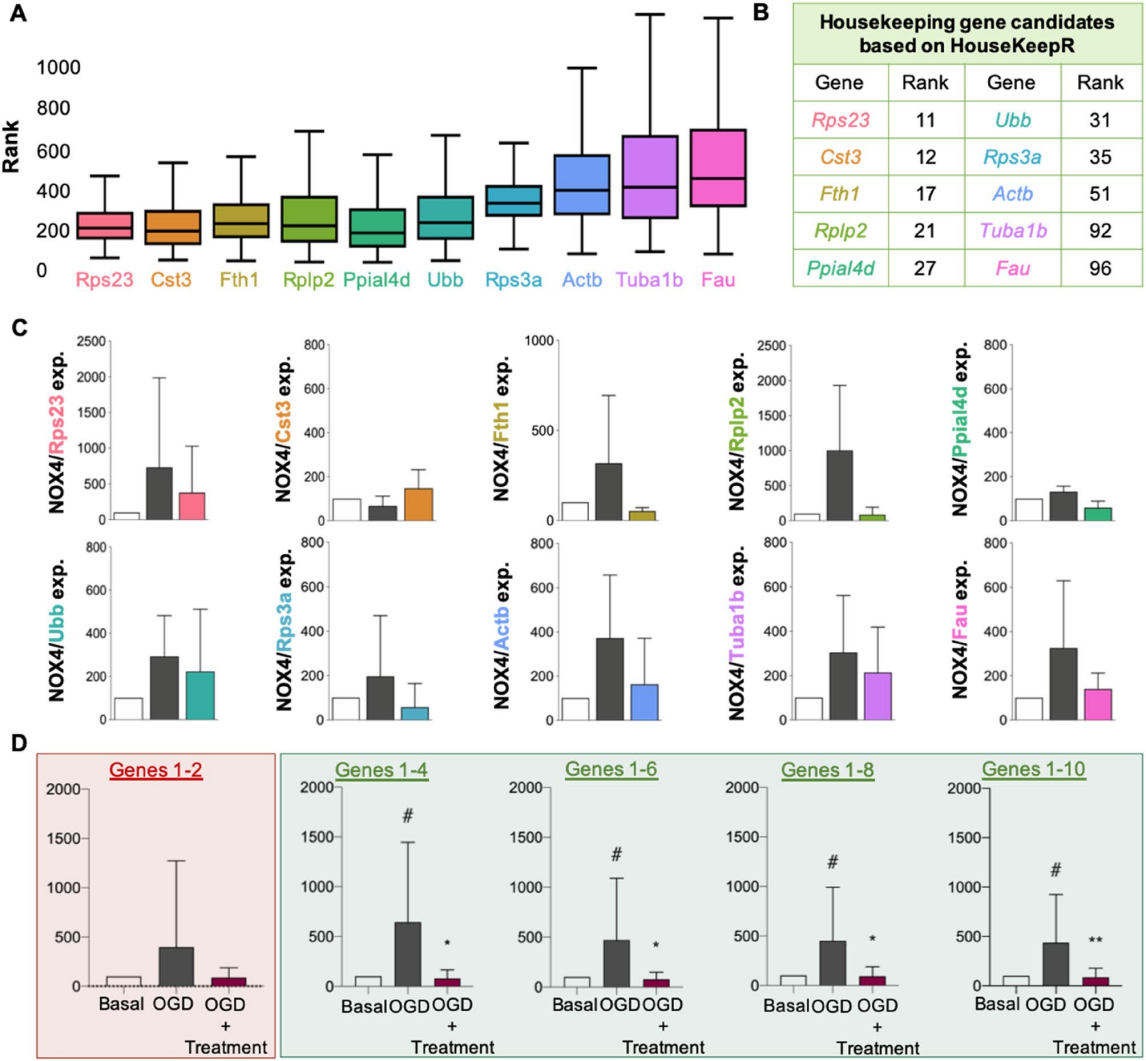
Un-biased de novo housekeeping gene panel generation and validation. (**A**) Ranking distribution of 10 randomly selected housekeeping gene candidates within the 100 top ranking generated over 10,000 bootstrap runs over all the samples in the 12 data sets selected for analysis. The order of the plot follows the final ranking in (**B**). (**B**) Final ranking of the selected normalisation gene candidates: Ubb, Fth1, Fau, Ppial4d, Cst3, Rps23, Actb, Tuba1, Rps3, Rplp2. This ranking is calculated by taking the mean bootstrap rank from A and penalising it for the absence of genes from each of the 12 data sets selected for analysis. (**C**) *Nox4* gene expression was normalised against the selected 10 genes of the ranking under 3 different experimental conditions, i.e. Basal, oxygen and glucose deprivation (OGD) and pharmacologically treated hippocampal tissue post-OGD. (**D**) 5 housekeeping genes panels were generated and validated: (i) Panel 1; 2 genes (*Ubb* and *Fth1*); (ii) Panel 2, 4 genes (*Ubb, Fth1, Fau* and *Ppial4d*); (iii) Panel 3, 6 genes (*Ubb, Fth1, Fau, Ppial4d, Cst3* and *Rps23*); (iv) Panel 4, 8 genes (*Ubb, Fth1, Fau, Ppial4d*, *Cst3, Rps23, Actb* and Tuba 1); (v) Panel 5, 10 genes (*Ubb, Fth1, Fau, Ppial4d, Cst3, Rps23, Actb, Tuba 1, Rps3* and *Rplp2*). Panel 2, 3, 4 and 5 showed stable *Nox4* gene expression results, while Panel 1 remained statistically not significant (^*#*^*p* < 0.05, n = 6; n = 6; *p < 0.05, n = 6; **p < 0.01, n = 6).

Once we demonstrated the reliability and reproducibility of the HouseKeepR tool, we performed an ultimate web lab validation step to confirm literature-based or find de novo and unbiased more suitable HK genes for this condition. We selected 12 data sets (Supplementary Table S3) associated with brain hypoxia/ischemia experiments in rats and mice (see Methods for details). HouseKeepR then calculated an overall ranking of 19,878 genes over 10,000 bootstrap samples. From the top 100 most stable genes, ten were randomly selected (#11-#96 in Fig. 4A,B). Since normalisation with multiple genes has been reported to outperform single-gene normalization^9^, we then assessed the reproducibility of *Nox4* induction under hypoxic conditions normalised against each of the ten genes individually (Fig. 4C) in a panel of the top two (#11 and #12, *Rps23* and *Cst3*), four, six, eight and ten (Fig. 4D). Importantly, only a panel of four HK genes ensured reliable normalisation and a statistically significant upregulation of *Nox4*. Reproducing differential *Nox4* expression in brain hypoxia based on single genes or a panel of two resulted in increased values that did not, however, reach statistical significance.

## Discussion

Choosing a reliably stable gene as HK gene is obviously critical for any differential gene expression study. Lack of stability can lead to false conclusions^26,27^. Here we demonstrate for both ex-vivo and in vivo models of brain hypoxia that literature-derived, repeatedly used HK genes display surprisingly considerable variance that can result in opposing conclusions on whether to consider a gene a differentially expressed or not. We demonstrate this for inducible *Nox4*, a promising therapeutic target in brain ischemia, currently under clinical testing (REPO-STROKE I and II)^17,22^.

The most widely used method for HK gene selection, i.e., literature-based and hence biased, may lead to reproducibility issues contributing to the quality crisis in biomedical research^28^. Thus, a systematic, unbiased method for HK gene selection is evidently needed. Moreover, for practical reasons, this method should be tissue- and condition-specific and user-friendly so biomedical scientists can widely adopt it without affording programming and coding skills. We have examined methods that select HK based on gene expression data and found limitations that hinder their adoption. RefGenes, for instance, which is part of the free version of the Genevestigator (https://genevestigator.com/) platform, relies on the standard deviation of HK genes to define housekeeping gene candidates. However, by focusing on a single gene expression data set, it neglects variations between conditions, which can be misleading in cases of low signal-to-noise ratio. While RefGenes offers a graphical user interface, it does not support exporting results for downstream analysis. NormFinder, which is available as Excel and R script, is another widely adopted method for validating HK genes in RT-qPCR data. In contrast to RefGenes, it considers not only the variance within a group of samples but also between groups, allowing for analysis across data sets, organisms, or conditions. While NormFinder is in principle applicable to microarray expression data, it is typically used to validate a set of candidate genes and is not suited for a genome-wide search for de novo HK gene candidates, where the runtime of the NormFinder algorithm becomes the limiting factor. Similarly, BestKeeper is available as an Excel spreadsheet that calculates the geometric mean of a set of input genes as an index which can be used to select the best combination of reference genes. The tool is limited, however, to 10 input genes and 100 samples while requiring expression values to be presented as Crossing Points^29^. Since existing tools do not satisfy one or more of the aforementioned criteria, we developed HouseKeepR, a web application for de novo HK gene discovery which fulfils, to our knowledge for the first time, all these criteria.

HouseKeepR suggests HK gene candidates that are highly reproducible both in silico and in vitro*.* Using our model system and target gene, *Nox4*, expression changes could be detected with single HK genes; the more subtle response of *Nox4* expression to pharmacological treatment was only detectable when using a panel of four HK genes for normalisation. Thus multiple HK gene-based normalisation results in more solid and uniform fold-changes correlations compared to using single gene expressions, providing consistency across data sets^5^. Thus, unbiased panel-based normalisation with condition-specific and cross-data set validated HK genes should become the future gold standard for normalising RT-PCR data. The number of HK genes for normalisation should be at least two (according to the MIQE guidelines)^30^, while the optimal number can be as high as 17, as shown for studying cellular senescence in human Endothelial Colony Forming Cells^31^. In this study, two reference genes did not achieve stable results, but a panel of at least four genes was necessary. Therefore, for a common experimental setup used in the biomedical field, we suggested that a panel of at least 2 to 4 would be required.

As a possibly perceived limitation, HouseKeepR is currently limited to microarray data in the GEO database. These data are not uniformly processed, and, hence, HousekeepR cannot expect data sets to be normalised for technical biases. Neglecting technical biases can lead to erroneous results when looking for a biological variation in, e.g., differential gene expression analysis. For HouseKeepR’s objective of finding suitable reference genes, however, it may even be advantageous to identify candidates that are robust to technical bias. Moreover, as RNA-seq data are becoming more prevalent, they can be incorporated as additional data sources, e.g., ARCHS4^32^ or GEMMA^33^. For large scale analysis, RNA-seq will eventually supersede qRT-PCR based gene expression analysis as the gold standard. However, HK gene-based normalisation will also need to be applied in this context^34,35^, and the need for unbiased and robust HK gene panels remains just as relevant.

## Methods

### Systematic review

A literature review focused on two in vitro models of brain ischemia: (i) hippocampal brain slices (HBS) and (ii) organotypic hippocampal culture (OHC) was performed. PubMed was searched for original papers and conference abstracts where these ex-vivo models appeared. No terms for RT-qPCR were included since PubMed only screens abstracts, titles and keywords, and RT-qPCR details are frequently mentioned only in the methods section. No language restriction was used. Our search strategy for the HBS model identified 364 records. First, these hits were screened based on title, abstract and results, excluding other, non-related ex-vivo models. Publications without RT-qPCR experiments or inaccessible full-text, i.e., only title/abstract published, were excluded. Finally, 16 articles were included for full-text screening. In 1 article, no normalisation gene was used, and in 4, RT-qPCR was conducted using other species. After a full-text assessment, 11 articles were considered. Similarly, our search strategy for model 2 (OHCs) identified 249 records in PubMed. Following the first screening, 38 articles were included for full-text screening. 2 of these did not use normalisation genes and 9 conducted RT-qPCR experiments using tissue from other species. Therefore, in total, 27 articles were considered for the OHC model. Studies were included if (i) the specific ex-vivo ischemia model was used; (ii) specific conditions were considered within the experiment; (iii) RT-qPCR experiments were conducted.

### Animals

Rats used for ex-vivo experiments were handled following the Guide for the Care and Use of Laboratory Animals and were previously approved by the Institutional Ethics Committee of Universidad Autónoma de Madrid, Spain, according to the European guidelines for the use and care of research animals by the European Union Directive of 22 September 2010 (2010/63/UE) and the Spanish Royal Decree of 1 February 2013 (53/2013). Similarly, in vivo experiments in mice strictly followed the Dutch law on animal experiments and were approved by the local animal experimental committee (Maastricht University, DEC2011-106). Both mice and rats were housed under controlled conditions of temperature (22 °C), humidity (55–65%), light (12 h light–dark cycles) and free access to water and standard laboratory chow. Male and female C57/Bl6 mice (8–12 weeks old), Sprague–Dawley adult rats (8–12 weeks old) and Sprague–Dawley pups (7–10 days old) were used.

### Ex-vivo acute model: preparation of hippocampal brain slices and induction of oxygen and glucose deprivation

Experiments were performed using hippocampal brain slices from adult male Sprague–Dawley rats (8–12 weeks old) as previously described in^36,37^. Briefly, rats were quickly decapitated, and forebrains were rapidly removed from the skull and placed into ice-cold Krebs bicarbonate dissection buffer (pH 7.4), containing (in mM): NaCl 120, KCl 2, CaCl_2_ 0.5, NaHCO_3_ 26, MgSO_4_ 10, KH_2_PO_4_ 1.18, glucose 11 and sucrose 200. At least 20 min before starting the experiment, chamber solutions were bubbled with either 95% O_2_/5% CO_2_ or 95% N_2_/5% CO_2_ gas mixtures to ensure O_2_ and N_2_ saturation, respectively. The hippocampus was quickly dissected and subsequently cut into transverse slices 300 μm thick using a Tissue Chopper Mcllwain. To recover from slicing trauma, slices were incubated in Krebs buffer for 45 min at 34 °C (stabilisation period). Then, control slices were incubated for 15 min in a Krebs-bicarbonate solution without sucrose (control solution). Oxygen and glucose deprivation was induced by incubating the slices for 15 min in a glucose-free Krebs-bicarbonate solution in which glucose was replaced by 2-deoxyglucose (OGD solution). Both solutions were pre-bubbled for 30 min with a 95% O_2_/5% CO_2_ or 95% N_2_/5% CO_2_, respectively. All experiments were performed at 37 °C. Following the OGD period, slices were returned to an oxygenated Krebs-bicarbonate solution containing glucose for 120 min (Re-Ox period). During the re-oxygenation period, either a pharmacological treatment (OGD + Treatment), or no extra measures (OGD) were taken. Specifically, treated hippocampal brain slices were exposed to 0.1 μM GKT136901 (NOX4 inhibitor). After the Re-Ox period, slices were collected and quickly shock-frozen.

### Ex-vivo chronic model: preparation of organotypic hippocampal slices and induction of oxygen and glucose deprivation

Hippocampal brain slices for cultures were obtained from brains of 7- to 10-days-old Sprague–Dawley rats. Organotypic cultures were prepared based on the methods previously described in^26^. Briefly, pups were quickly decapitated and brains removed from the skull and dissected. The hippocampus was cut into 300 μm-thick slices using a Tissue Chopper Mcllwain. Then, they were separated in sterile ice-cold Hank’s balanced salt solution (HBSS, Biowest, Madrid, Spain) containing (in mM): glucose 15, CaCl_2_ 1.3, KCl 5.36, NaCl 137.93, KH_2_PO_4_ 0.44, Na_2_HPO_4_ 0.34, MgCl_2_ 0.49, MgSO_4_ 0.44, NaHCO_3_ 4.1, HEPES 25, 100 U/ml penicillin, and 0.100 mg/ml gentamicin. Six slices were placed on each Millicell-0.4 μm culture insert (Millipore, Madrid, Spain) within each well of a six-well culture plate. Specific neurobasal medium (Invitrogen, Madrid, Spain) enriched with 10% of fetal bovine serum (Sigma-Aldrich, Madrid, Spain) was used for the next 24 h (1 ml/well). 24 h later, B27 supplement and antioxidants were added to the culture medium. Slices were in culture for 4d before inducing the OGD period. On day 6, inserts were placed in 1 ml of OGD solution composed of (in mM): NaCl 137.93, KCl 5.36, CaCl_2_ 2, MgSO_4_ 1.19, NaHCO_3_ 26, KH_2_PO_4_ 1.18, and 2-deoxyglucose 11 (Sigma-Aldrich, Madrid, Spain). The cultures were then placed in an airtight chamber (Billups-Rothenberg Inc., USA) and exposed for 5 min to 95% N_2_/5% CO_2_ gas flow to ensure oxygen removal. Then, the chamber was sealed for 15 min and placed at 37 °C (OGD period). At the same time, control cultures were maintained under a normoxic atmosphere in a solution with the same composition as previously described but containing glucose (15 mM) instead of 2-deoxyglucose. Pharmacological treatment was added to the cultures before returning them to normal oxygen and glucose concentrations for 24 h (re-oxygenation period). Specifically, treated organotypic brain slices were exposed to either a single treatment or a combination of 0.1 μM GKT136901 (NOX4 inhibitor) after 15 min of OGD. After the Re-Ox period, slices were collected and quickly shock-frozen.

### In vivo model: transient occlusion of the middle cerebral artery (tMCAO) in mice

Stroke surgery was conducted as previously described in^38^. After administering a painkiller, animals were anesthetised with isoflurane (induction 4–5% in air, maintenance 2–2.5% in air) and placed on a heating pad that maintained the rectal temperature at 37.0 °C using a feedback-controlled infrared lamp. Using a surgical microscope (Wild M5A, Wild Heerbrugg, Gais, CH), a midline neck incision was made, and both the right common and external carotid arteries were isolated and permanently ligated while a microvascular clip was temporarily placed on the internal carotid artery. A small incision into the common carotid artery was performed where the silicon rubber-coated 6.0 nylon monofilament (602312PK10, Doccol Corporation, Sharon, MA, USA) was inserted until resistance was felt. The monofilament tip should be specifically located intracranially at the origin of the right middle cerebral artery, thereby interrupting blood flow. The filament was fixed with a tourniquet suture to prevent dislocation. 1 h after occlusion of the middle cerebral artery, reperfusion was initiated by monofilament removal. Wounds were carefully sutured, and animals could recover in a temperature-controlled cupboard. Pharmacological treatment (GKT136901, 10 mg/kg) was given via i.p. injections, 2 h and 12 h after the start of ischemia. Animals were sacrificed 24 h after induction of ischemia by cervical dislocation. Brains were quickly removed, and shock froze.

### RNA extraction, quantification and reverse transcription

Hippocampal brain slices and brain tissue from ex-vivo and in vivo models were crushed and homogenised using TRI Reagent^®^ (Sigma-Aldrich, The Netherlands). 100 μl of chloroform was added to the samples, followed by a 15 min centrifugation at 11,000 rpm and 4 °C. After centrifugation, 250 μl of isopropanol was added to the upper phase (mRNA) and then kept for 1 h at − 20 °C. After incubation, samples were centrifuged during 10 min at 13,000 rpm, and 4 °C. 200 μl ethanol 80% was added to the supernatant followed by 10 min centrifugation at 13,000 rpm. After ethanol removal, the mRNA was dissolved in RNAse free water. mRNA was quantified spectrophotometrically using the Nanodrop 2000 device. 0.08 µg of total mRNA was reverse transcribed to cDNA with the High-Capacity Reverse Transcription Kit (Applied Biosystems, The Netherlands) according to the manufacturer’s protocol.

### Real-time PCR

mRNA levels of studied genes were quantified using the fluorescent Taqman^®^ technology. We used TaqMan^®^ gene expression arrays (TaqMan^®^ Universal PCR Master Mix, ThermoFisher Scientific, The Netherlands) for all species: (i) For rat: *β2-microglobulin* (Rn00560865_1, ThermoFisher Scientific, The Netherlands), *β-actin* (Rn00667869_m1, ThermoFisher Scientific, The Netherlands), *Rpl13* (Rn00821946_m1, ThermoFisher Scientific, The Netherlands), *18S* (Hs99999901_s1, ThermoFisher Scientific, The Netherlands), *Hprt* (Rn01527840_m1, ThermoFisher Scientific, The Netherlands), *Sdha* (Rn00590475_m1, ThermoFisher Scientific, The Netherlands), *Ywhaz* (Rn00755072_m1, ThermoFisher Scientific, The Netherlands), *Gapdh* (Rn01775763_g1, ThermoFisher Scientific, The Netherlands), *Nox4* (Rn01506793_m1, ThermoFisher Scientific, The Netherlands). (ii) For mice: *β2-microglobulin* (Mm00437762_m1, ThermoFisher Scientific, The Netherlands), *β-actin* (Mm02619580_g1, ThermoFisher Scientific, The Netherlands), *Rpl13* (Mm02526700_g1, ThermoFisher Scientific, The Netherlands), *18S* (Hs99999901_s1, ThermoFisher Scientific, The Netherlands), *Hprt* (Mm03024075_m1, ThermoFisher Scientific, The Netherlands), *Sdha* (Mm01352366_m1, ThermoFisher Scientific, The Netherlands), *Ywhaz* (Mm03950126_s1, ThermoFisher Scientific, The Netherlands), *Gapdh* (Mm99999915_g1, ThermoFisher Scientific, The Netherlands), *Ppia4d* (Mm01191872_g1, ThermoFisher Scientific, The Netherlands), *Fth1* (Mm00850707_g1, ThermoFisher Scientific, The Netherlands), *Cst3* (Mm00438347_m1, ThermoFisher Scientific, The Netherlands), *Rps23* (Mm03019701_g1, ThermoFisher Scientific, The Netherlands), *Rplp2* (Mm00782638_s1, ThermoFisher Scientific, The Netherlands), *Ubb* (Mm01622233_g3, ThermoFisher Scientific, The Netherlands), *Fau* (Mm02601595_u1, ThermoFisher Scientific, The Netherlands), *Tuba1* (Mm00846967_g1, ThermoFisher Scientific, The Netherlands), *Rps3* (Mm00656272_m1, ThermoFisher Scientific, The Netherlands) (Table S2). Water controls were included to ensure specificity, and the comparative 2^−ΔΔCt^ method was used for relative quantification of gene expression. When pre-designed primers are not commercially available, manual design is strongly recommended based on the required experimental scenario. In case of multiple comparisons, experimental biological samples were normalised to either 1 specific gene or different ones of the proposed housekeeping panel with the same final number of normalisation rounds.

### NormFinder stability score

The NormFinder algorithm^12^ can be used to assess the stability of normalisation genes across groups based on RT-qPCR measurements. Briefly, NormFinder models the variation within and between sample groups to estimate a HK gene candidate’s expression stability in the form of a distribution

$${{\mathrm{f}}_{\mathrm{ig}}=\mathrm{z}}_{\mathrm{ig}}- {\uptheta }_{\mathrm{g}}- {\mathrm{\alpha }}_{\mathrm{i}}$$

where ${\mathrm{z}}_{\mathrm{ig}}$ is the mean, log-transformed expression level of the gene $\mathrm{i}$ for all samples in group $\mathrm{g}$, ${\uptheta }_{\mathrm{g}}$ is the average amount of mRNA for group $\mathrm{g}$, and ${\mathrm{\alpha }}_{\mathrm{i}}$ is the mean expression level for gene $\mathrm{i}$ over all groups. Hence, the resulting distribution is an additive measure of variance within and between groups. NormFinder transforms the resulting stability distribution to an easier-to-interpret stability value $\rho \_ig$ by taking the absolute value of the mean + 1 standard deviation:

$${\rho }_{ig}=\left|\mathrm{mean}\left({\mathrm{f}}_{\mathrm{ig}}\right)+\mathrm{sd}\left({\mathrm{f}}_{\mathrm{ig}}\right)\right|$$

### HouseKeepR method

Normalisation genes should show stable expression levels across tissues and conditions of interest, i.e., a low variance and log fold change are desirable. This first characteristic is captured by the coefficient of variation (CV):

$$CV=\frac{Standard\, Deviation}{Mean\, Expression}$$

The CV captures the relation of standard deviation and mean expression and is thus a unitless measure of noise and overdispersion commonly used for describing the robustness of a measurement^39^. As a second characteristic, we consider the fold change between conditions to avoid selecting normalisation genes affected by the condition of interest. As was previously shown, the CV tends to be higher for genes with low expression and plateaus for higher expression levels^40^. In addition, fold changes tend to be inflated for lowly expressed genes^41^. Thus, ranking normalisation genes by both CV and fold change will help select genes with comparably high expression that are not affected by measurement bias and robustly expressed between conditions. We compute both the CV and the fold change based on raw expression values for each gene across the samples of each data set. Then the fold change is inverted if it is < 1 to get the absolute value of change. Since most GEO data sets are provided as log-transformed expressions, exponentiation is applied to retrieve raw values. To combine CV and fold change into a single quality score, we first rank fold changes and CVs independently before computing a joint rank product. An ideal normalisation gene candidate consequently achieves a low rank across various data sets. To avoid that the selected HK genes exhibit high-rank stability by chance, we use bootstrapping, i.e. sampling with replacement. As the bootstrap samples will always have a different composition, we can judge the effect of sampling bias on rank stability. For each bootstrap repetition, the genes are ranked by the mean rank across the sampled data sets. Genes with stable ranks over a large number of bootstrap repetitions, e.g., 10,000, are selected as the top-performing normalisation gene candidates. The combined use of ranks and bootstrapping enables the processing of different array data sets without the need for batch effects removal or cross-platform normalisation, enabling HouseKeepR to perform largely automated meta-analyses for normalisation gene identification.

### HouseKeepR R shiny web interface

Existing tools such as NormFinder and BestKeeper do not offer a user-friendly interface and do not afford genome-wide coverage. RefGenes has a user interface but is not capable of integrative analysis across publicly available gene expression data, leading to unstable results when using different expression platforms (Supplementary Fig. S7). This motivated us to make the HouseKeepR method easily accessible to the community via an R shiny web interface which leads users through the process of de-novo detection of suitable normalization genes for user-selected tissues and conditions. The basis for this analysis is the Gene Expression Omnibus (GEO)^42^, a widely used repository currently offering access to more than a hundred thousand gene expression profiles of more than three million samples. HouseKeepR allows users to directly query GEO for data sets related to specific organisms, conditions and tissues. HouseKeepR shows the query's result and provides additional information about available studies. Next, users can select studies relevant to de-novo HK gene discovery and assign conditions and control labels for each sample. Once the user starts an analysis, HouseKeepR will (i) download expression data using the ‘GEOquery’ R package^43^, (ii) map microarray probe identifiers to gene identifies such as Entrez or Ensembl, (iii) identify and map gene homologs across organisms, and (iv) compute the differential expression between condition and control samples using the ‘limma’ Bioconductor package^44^ primer. Once the analysis is complete, HouseKeeepR reports the top-performing HK genes together with informative visualisations of their ranks across data sets. Figure 3 provides an overview of these steps. HouseKeepR is released under an open-source license (https://github.com/biomedbigdata/housekeepr) and available for local use as a docker container or an online web application at https://exbio.wzw.tum.de/housekeepr.

### HouseKeepR application to mouse and rat models of ischemic stroke

We demonstrate the practical application of HouseKeepR by validating de-novo identified HK genes with RT-qPCR. To this end, we selected rats and mice as organisms, ischemia, ischemic or stroke as conditions and brain as tissue. These parameters returned 163 data sets, from which we excluded those that did not satisfy our model criteria or that showed special model attributes that could affect predictions of normalisation genes, such as genetically modified organisms. Finally, 12 high-quality data sets (Supplementary Table 1) were selected for further analysis. An AIMe report^45^ for reproducible machine learning was created and deposited at https://aime.report/F9IKDV.

### Statistical analysis

Experimental results were presented as means ± SEM. Differences between groups were determined by applying a one-way ANOVA followed by two-way ANOVA followed by Dunnett’s Multiple Comparison test or Student’s two-tailed t-test and Mann–Whitney test experiments when appropriate. For repeated measurements, a two-way ANOVA was used. Statistical analysis was conducted using GraphPad Prism version 5.00. The level of statistical significance was set at p < 0.05.

## Supplementary Information

Supplementary Information.

## Data Availability

The datasets used and/or analysed during the current study are available from the corresponding author on reasonable request.
