# Supplementary material for: Un-biased housekeeping gene panel selection for high-validity gene expression analysis
Source: Sci Rep. 2022 Jul 19;12:12324. doi: 10.1038/s41598-022-15989-8 (PMC9296577; doi:10.1038/s41598-022-15989-8)
Supplement: Supplementary file 1 — Supplementary Information. [file 41598_2022_15989_MOESM1_ESM.pdf]

Supplementary Information

Un-biased housekeeping gene panel selection for high-validity gene expression analysis

Ana I. Casas[1,2,+,*], Ahmed A. Hassan[2,+], Quirin Manz[3,+], Christian Wiwie[4], Pamela Kleikers[2], Javier Egea[5], Manuela G. López[6], Markus List[7,†], Jan Baumbach[3,†], and Harald H.H.W. Schmidt[2,†,*]

[1]Department of Neurology, University Clinics Essen, Essen, Germany

[2]Department of Pharmacology & Personalised Medicine, MeHNS, Faculty of Health, Medicine and Life Sciences, Maastricht University, Maastricht, The Netherlands

[3]Faculty of Mathematics, Informatics and Natural Sciences, University of Hamburg, Hamburg, Germany

[4]Department of Mathematics and Computer Science, University of Southern Denmark, Odense, Denmark

[5]Instituto de Investigación Sanitaria, Servicio de Farmacología Clínica, Hospital Universitario de la Princesa, Madrid, Spain

[6]Instituto de I+D del Medicamento Teófilo Hernando (ITH), Departamento de Farmacología, Facultad de Medicina, Universidad Autónoma de Madrid, Madrid, Spain

[7]Chair of Experimental Bioinformatics, TUM School of Life Sciences Weihenstephan, Technical University of Munich, München, Germany

[+]Ana I. Casas, Ahmed A. Hassan, Quirin Manz contributed equally to this work

[†]Markus List, Jan Baumbach, Harald H.H.W. Schmidt contributed equally to this work

[*]Email: anaisabel.casasguijarro@uk-essen.de and hschmidt@ppmlab.net

Supporting information

Supplementary Figures

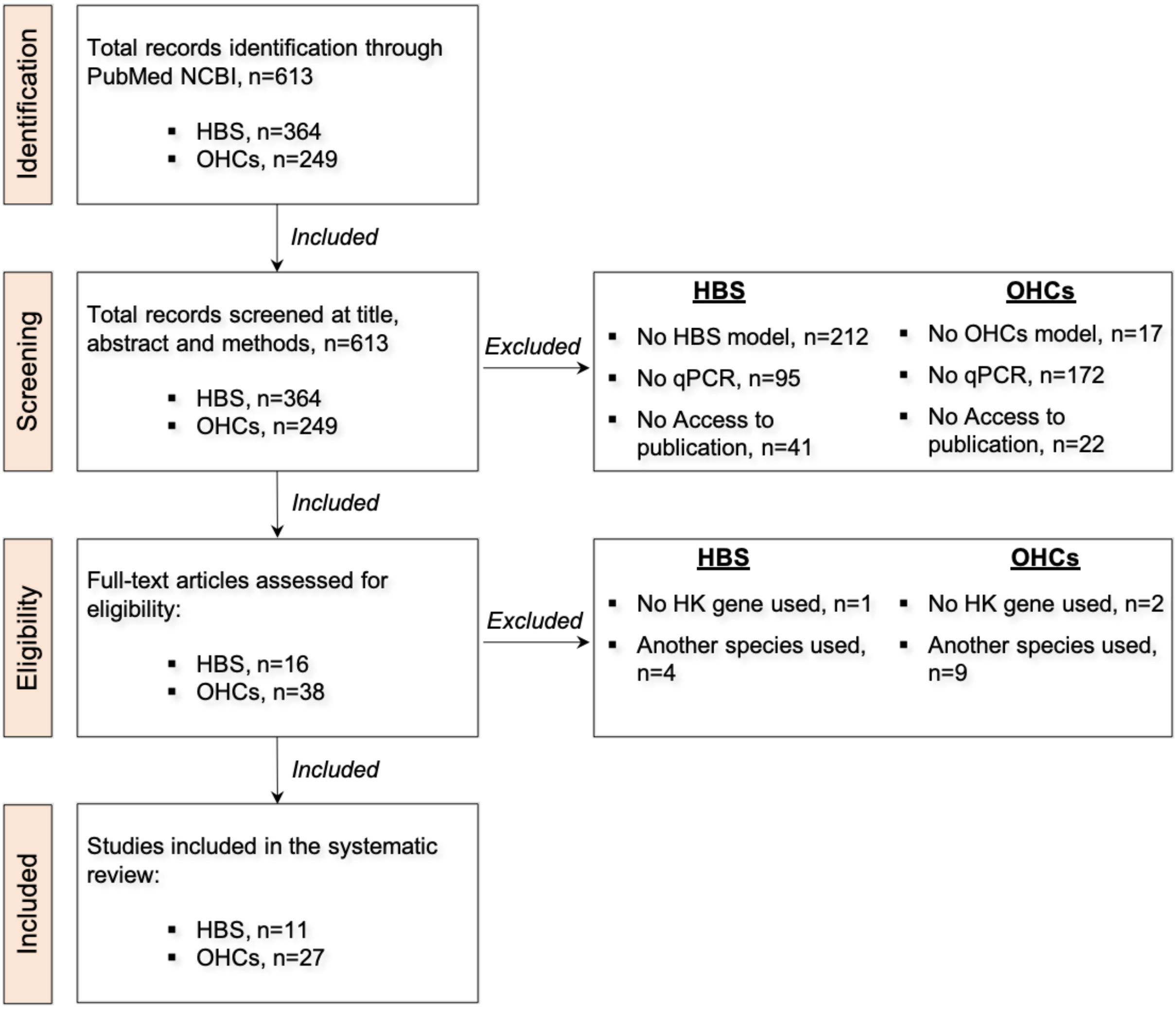

Supplementary Fig. 1. Flow diagram of the housekeeping based systematic review. First, we proceed with suitable literature identification followed by partial screening (title and abstract), full text screening (results and methods sections), and final implementation of previously decided exclusion criteria (listed in the right boxes above).

Gene Symbol(s)	Ensembl ID(s)	#Data sets	Rank Mean	Rank Variance	Rank
Mt-co1	ENSRNOG00000034234	11/12	5.3131	2.9023	1
Ubb	ENSRNOG00000042271	9/12	9.1297	3.272	2
Mt-atp6	ENSRNOG00000031979	7/12	9.5614	178.4173	3
Mt-co2	ENSRNOG00000030371	11/12	15.4169	5.6141	4
Fth1	ENSRNOG00000022619	11/12	20.1958	9.1929	5
LOC687780, Fau, LOC100360647	ENSRNOG00000020982, ENSRNOG00000046393, ENSRNOG00000047365	11/12	26.5895	8.3883	6
Ppial4d	ENSRNOG00000055068	6/12	27.4902	215.5905	7
Mt-co3	ENSRNOG00000030700	7/12	28.4778	178.9081	8
Mt-nd3	ENSRNOG00000033615	8/12	30.2172	10.7486	9
Cst3	ENSRNOG00000005195	12/12	30.6617	17.5866	10
Mt-nd1	ENSRNOG00000030644	10/12	30.7543	23.1536	11
Rps23	ENSRNOG00000016580	12/12	43.1639	12.6347	12
Mt-cyb	ENSRNOG00000031766	11/12	49.8794	26.3948	13
Actb	ENSRNOG00000034254	12/12	51.8344	39.2029	14
Tuba1b	ENSRNOG00000053468	10/12	60.3932	16.589	15
Rps3a	ENSRNOG00000011893	11/12	66.8536	19.6935	16
LOC108352650, LOC108350501, LOC108351482, Rps29	ENSRNOG00000004196, ENSRNOG00000028939, ENSRNOG00000029443, ENSRNOG00000032542	12/12	68.0881	46.319	17
Rplp2, LOC100911575	ENSRNOG00000002116, ENSRNOG00000037607	12/12	68.2227	19.1185	18
LOC108351137	ENSRNOG00000018630	12/12	72.3901	25.4942	19
Tuba1a	ENSRNOG00000060728	10/12	75.1609	32.0659	20

Supplementary Fig. 2. HouseKeepR candidate genes ranking. The final ranking as shown in the HouseKeepR application after the analysis has finished. This ranking shows the HGNC gene symbols as well as their corresponding Ensembl ID. Multiple splice variants are mapped to the same row. Indicated is also the number of data sets where this gene was found among the selected expression data sets, the mean and variance of ranks derived from the bootstrapping of all samples, and finally, the ranking of the HK genes within the current analysis.

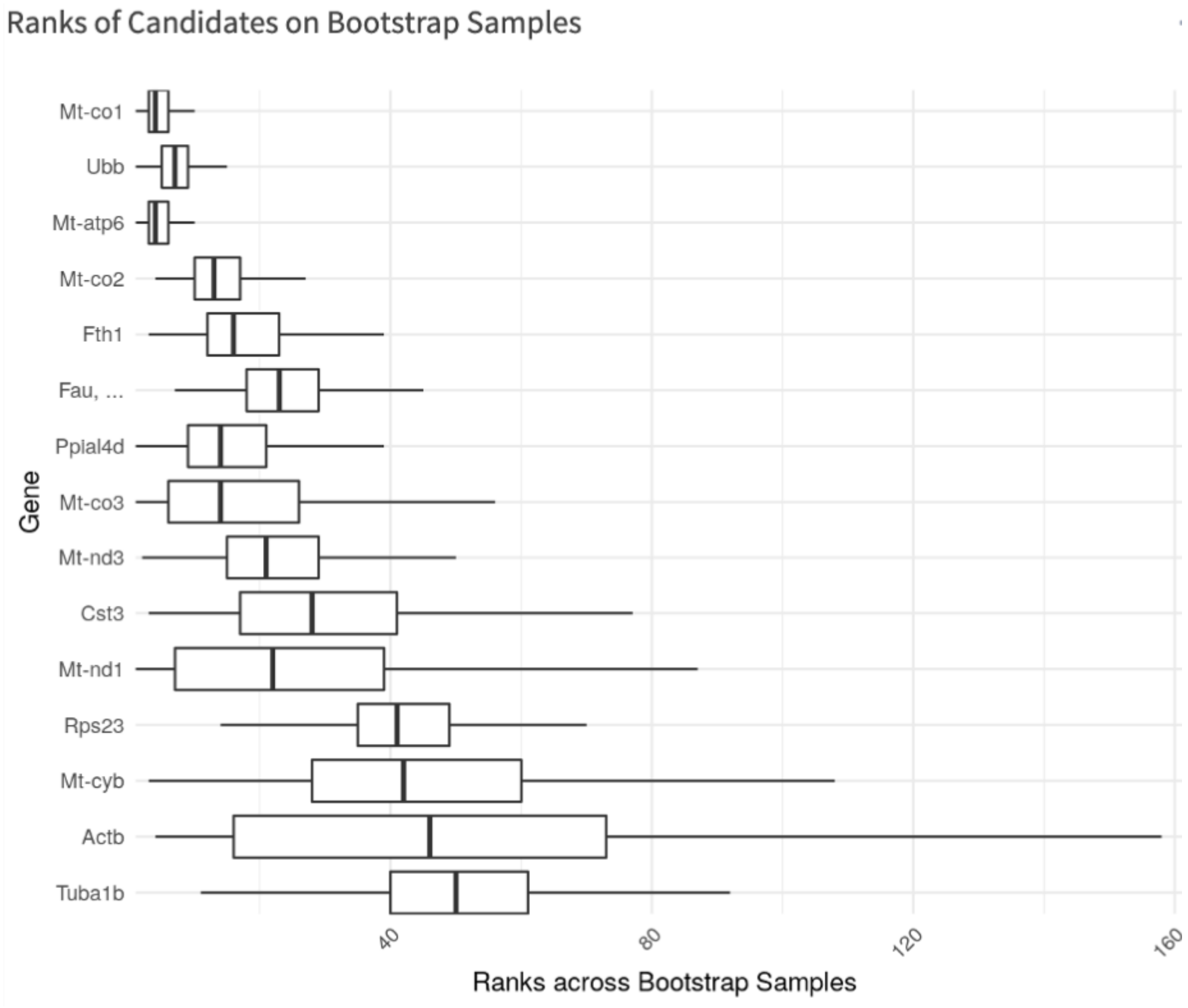

Supplementary Fig. 3. HouseKeepR gene ranking distribution over samples. To help with the explicability of results, HouseKeepR shows a box plot presenting the distribution of HK candidate genes ranking over all the samples of the chosen data sets. The plot shows the median, first and third quartiles of the ranking for each candidate gene, while not taking into account the penalization based on missing samples. Since outliers could occur often when bootstrapping, a checkbox is added to hide them.

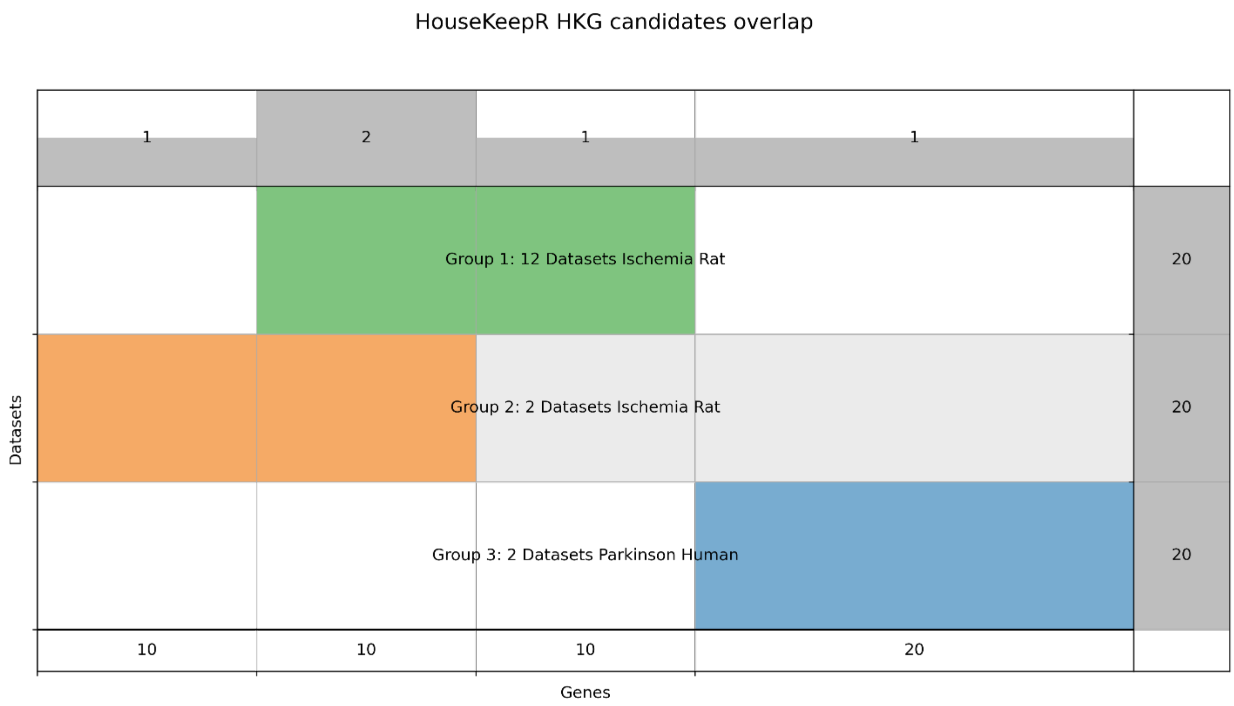

Supplementary Fig. 4. Overlap between the top 20 gene candidates generated by HouseKeepR on 3 different groups of data sets. The 3 different groups illustrate the stability and reliability of HouseKeepR predictions. Groups 1 and 2 share the same tissue, condition and organism, i.e., brain, ischemia and rat, while group 3 shares only the tissue with the other, i.e. brain, Parkinson and human. Group 1 and 2 overlap in 10 out of 20 genes, while there is no overlap with group 3.

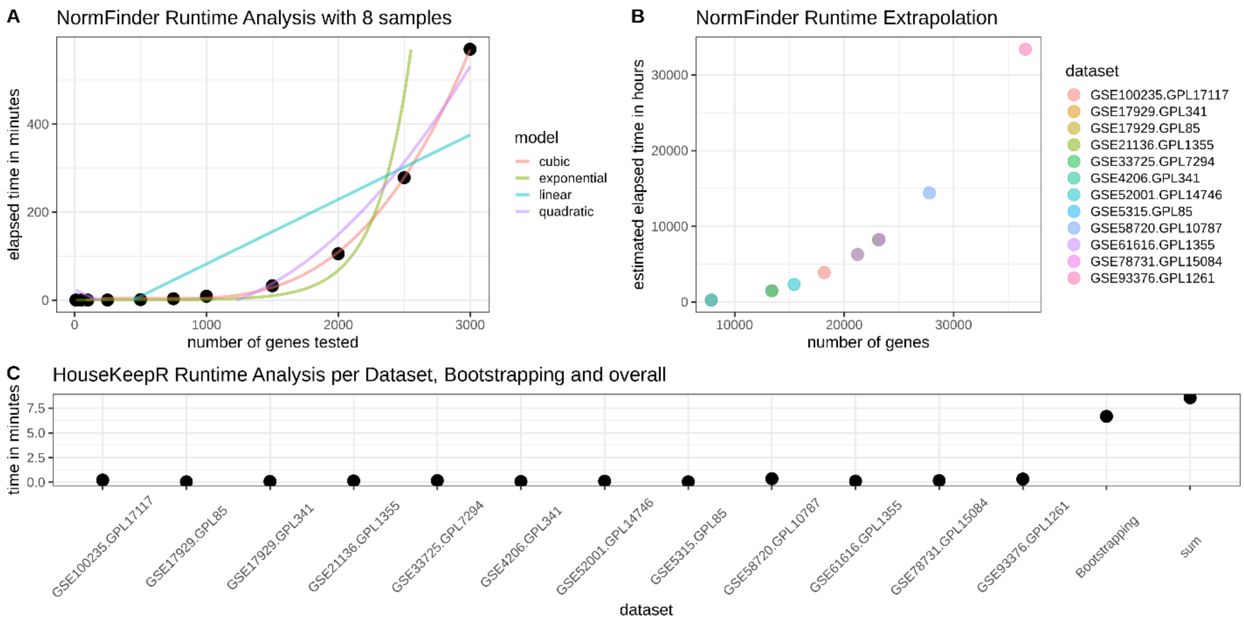

Supplementary Fig. 5. Runtime comparison between NormFinder and HouseKeepR. (A) NormFinder analysis on a small data set with 8 samples and up to 3,000 genes revealing a cubic increase in runtime. (B) Extrapolation of the runtime for the data sets selected in this study. (C) Runtime of the HouseKeepR approach including bootstrapping for the same data sets as well as the overall runtime.

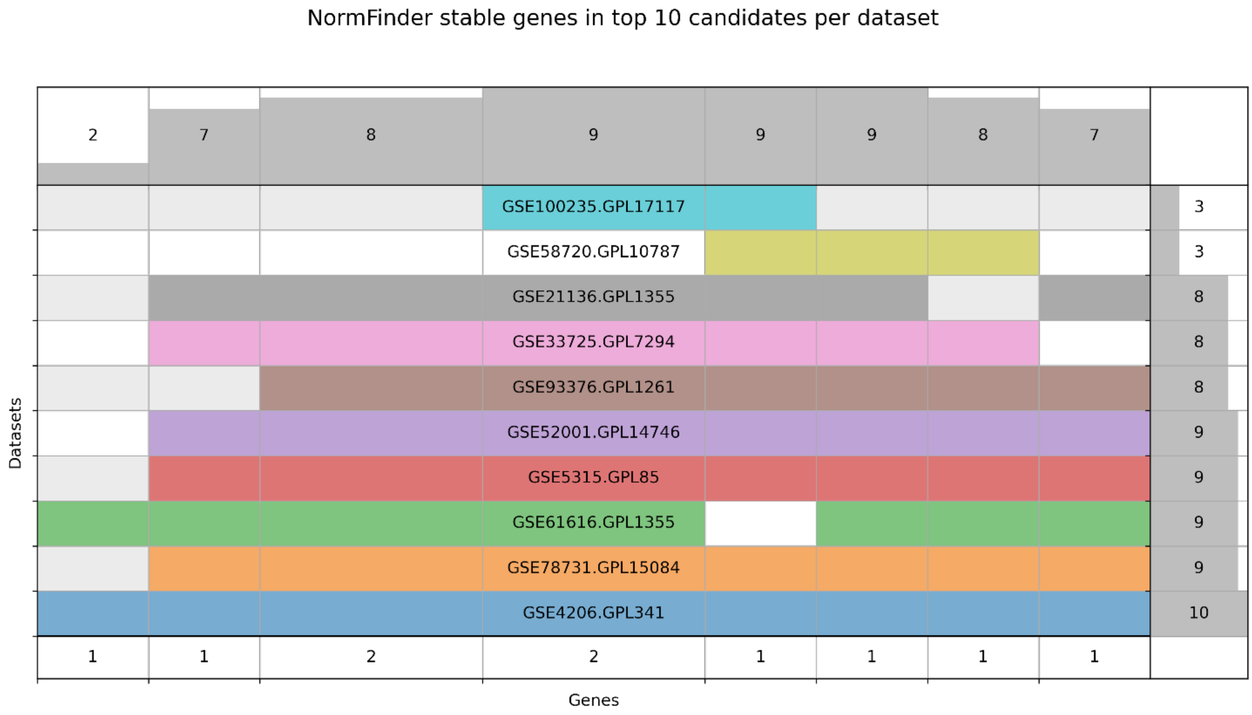

Supplementary Fig. 6. Stability of top 10 HouseKeepR HKG candidates evaluated by NormFinder. For validating the accuracy of HouseKeepR housekeeping genes, the stability of genes was calculated within each data set using NormFinder algorithm. Stable genes were considered to be with a stability score of 0.15 or lower. The figure shows how many genes out of the top 10 HouseKeepR candidates are stable (x-axis subtotals) as well as the overlap of stability across the different data set used in the analysis (y-axis subtotals).

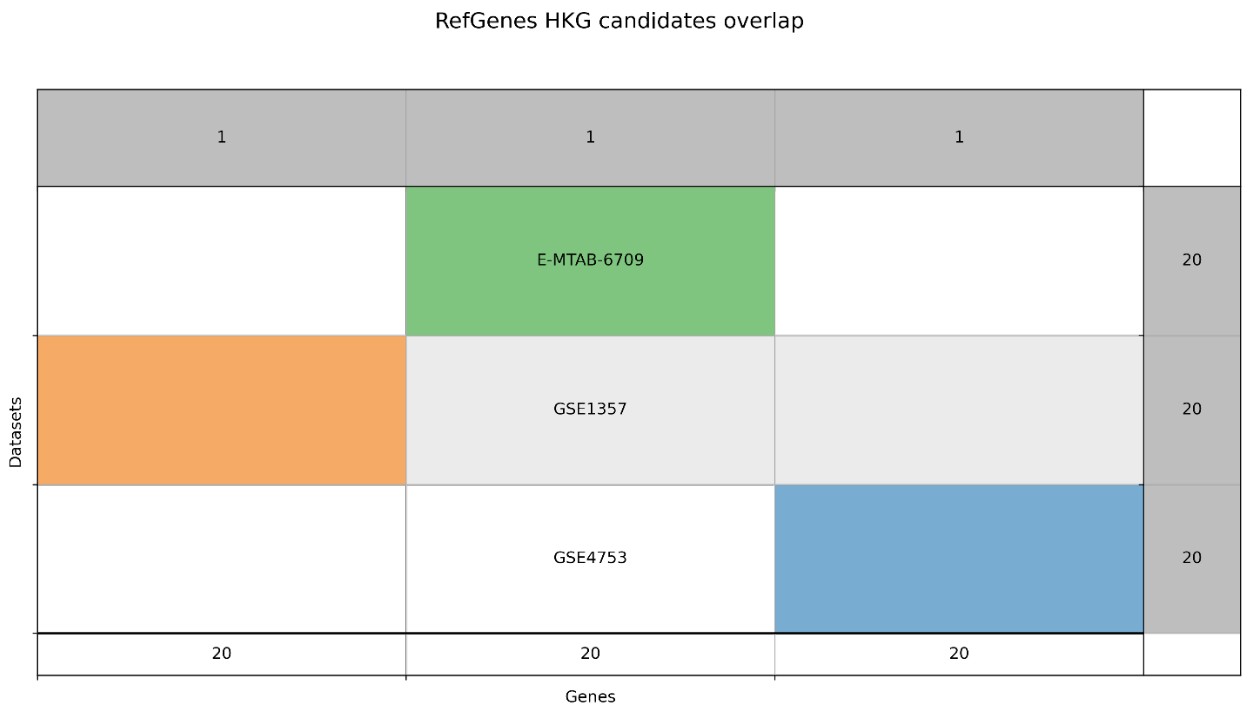

Supplementary Fig. 7. Overlap between the top 20 gene candidates generated by RefGenes on three different data sets. The figure shows three different data sets with different underlying platforms (E-MTAB-6709 is an RNA-seq platform, while GSE1357 and GSE4753 are two different Affymetrix microarray platforms). There is no overlap between the gene candidates for each data set since RefGenes relies only on standard deviation of expression levels, which require normalization between platforms.

Supplementary Tables

Supplementary Table 1. Search strategy systematic review

Model	Search term
HBS	(OGD OR oxygen-glucose deprivation OR oxygen glucose deprivation OR combined oxygen and glucose deprivation OR oxygen-glucose deprivation and reoxygenation OR oxygen and glucose deprivation) AND (hippocampal brain slices OR rat hippocampal brain slices OR rodent hippocampal brain slices OR acute hippocampal slices OR acute hippocampal brain slices)
OHCs	(OGD OR oxygen-glucose deprivation OR oxygen glucose deprivation OR combined oxygen and glucose deprivation OR oxygen-glucose deprivation and reoxygenation OR oxygen and glucose deprivation) AND (organotypic brain slices OR organotypic brain slice culture OR rodent hippocampal slice cultures OR rodent organotypic hippocampal slice cultures OR OHSCs OR organotypic hippocampal slice culture OR organotypic hippocampal slices OR organotypic hippocampal culture)

Supplementary Table 2. Candidate Reference genes

Gene symbol	Gene name	TaqMan assay number - Rat	TaqMan assay number - Mice
β*2-microglobulin*	*β2-microglobulin*	Rn00560865	Mm00437762
β *−actin*	*β-actin*	Rn00667869	Mm02619580
Rpl13	*Ribosomal Protein L13A*	Rn00821946	Mm02526700
18S	*Eukaryotic 18S rRNA*	Hs99999901	Hs99999901
Hprt	*Hypoxanthine phosphoribosyltransferase*	Rn01527840	Mm03024075
Sdha	*Succinate dehydrogenase complex, subunit A*	Rn00590475	Mm01352366
Ywhaz	*Tyrosine 3-monooxygenase*	Rn00755072	Mm03950126
Gadph	*Glyceraldehyde-3-phosphate dehydrogenase*	Rn01775763	Mm99999915
Ubb	*ubiquitin B*	-	Mm01622233
Fau	*FAU ubiquitin like and ribosomal protein S30 fusion*	-	Mm02601595
Fth1	*Ferritin heavy chain 1*	-	Mm00850707
Cst3	*Cystatin 3*	-	Mm00438341
Pp1a4d	*Peptidylprolyl isomerase (cyclophilin)-like 4*	-	Mm01191872
Rps23	*Ribosomal protein 23*	-	Mm03019701
Tuba1b	*tubulin alpha 1b*	-	Mm00846967
Rplp2	*Ribosomal protein lateral stalk subunit P2*	-	Mm00782638
Rps3	*Ribosomal protein S3*	-	Mm00656272

Supplementary Table 3. Selected data sets for HouseKeepR ischemia analysis

GEO Data set Accession	Platform ID
GSE100235	GPL17117
GSE17929	GPL341
GSE17929	GPL85
GSE21136	GPL1355
GSE33725	GPL7294
GSE4206	GPL341
GSE52001	GPL14746
GSE5315	GPL85
GSE58720	GPL10787
GSE61616	GPL1355
GSE78731	GPL15084
GSE93376	GPL1261
